# Quality-Oriented Smart Sensing and Sensing-to-Control Pathways Across the Mechanized Forage Production Chain: A Review

**DOI:** 10.3390/s26206375

**Published:** 2026-10-09

**Authors:** Dejiang Liu, Tao Sun, Wenxiang Zhang, Zeyu Liu, Shimin Ma, Chengyi Zhong, Keheng Yao

**Affiliations:** 1 Nanjing Institute of Agricultural Mechanization, Ministry of Agriculture and Rural Affairs, Nanjing 210014, China; liudejiang@caas.cn (D.L.);; 2 Nanjing Institute of Vegetable Science, Nanjing 210014, China

**Keywords:** forage mechanization, smart sensing, quality-oriented control, sensor fusion, adaptive control, production-chain coordination

## Abstract

The increasing automation of mechanized forage production has improved operational efficiency, but achieving consistent product quality remains challenging because material conditions evolve continuously across sequential operations. Current control strategies primarily focus on machine-level objectives, such as load regulation, throughput stabilization, and actuator response, while the linkage between operational decisions and final forage quality is still insufficiently established. Machinery-, sensor-, and stage-centered reviews chiefly catalog technologies and prediction performance, without consistently testing whether observations are representative, transferable, timely, and assigned to actionable material. This critical narrative review integrates recent evidence identified through Web of Science Core Collection and CNKI searches, supplemented by citation tracing of foundational studies. It presents a state-centered framework for smart sensing and sensing-to-control pathways throughout the mechanized forage production chain. Key intermediate states, including moisture distribution, leaf integrity, windrow geometry and linear density, mass flow, particle characteristics, bale density, and pore structure, are analyzed as the links between mechanical actions and downstream outcomes, such as drying performance, material loss, fermentation, heating risk, and storage stability. Sensing technologies based on electrical, mechanical, optical, microwave, weighing, and spectroscopic principles are evaluated from the perspectives of measurement representativeness, transferability, latency, uncertainty, and operational validity rather than prediction accuracy alone. Prediction accuracy describes agreement with a reference, whereas decision utility reflects whether a valid, representative, and timely estimate can change an operational decision and reduce downstream quality risk. The reviewed evidence indicates that practical quality-oriented automation requires rapid proxy-state feedback for immediate machine regulation combined with batch-linked quality measurements for delayed verification and model updating. Future progress depends on establishing causal machine–quality relationships, transferable sensing models, cross-stage data continuity, and production-scale validation.

## 1. Introduction

Forage yield, nutritive value, and continuity of supply shape the cost and stability of ruminant production. Mechanization is moving beyond labor substitution by stand-alone machines toward coordinated operations, online state sensing, and adaptive control. The value of that transition is determined by material and quality preservation across successive operations, not by the capacity or field efficiency of an individual machine [1,2,3,4].

Intelligent mechanization is difficult because harvested forage is moist, heterogeneous, deformable, and composed of organs with contrasting geometry, mechanics, moisture, and nutritive composition. Respiration, moisture redistribution, oxidation, and microbial activity continue after mowing. The material entering each operation therefore reflects crop development, weather exposure, and prior handling, making production intrinsically path-dependent [5].

Mechanical action both enables preservation and creates damage. Cutting, conditioning, tedding, raking, pickup, chopping, baling, wrapping, and drying alter tissue integrity, moisture transport, material flow, particle structure, density, and pore architecture [6]. A locally efficient action can create an unfavorable state for the next stage; final quality is the cumulative result of these coupled mechanical and biological transitions.

Online measurements can recover moisture, mass flow, windrow geometry, composition, machine load, and bale state [3,7,8,9,10,11]. Measurement alone is insufficient, however. An observation has to represent the moving material, be assigned to the correct location or batch, remain valid under the current machine–material–environment conditions, and arrive before corrective action is no longer effective. Calibration accuracy does not establish this measurement-to-actuation readiness. The same sensor may observe a different material portion from the one affected by the actuator because forage is transported, mixed, and compressed between sensing and action locations. Response time, transport delay, and spatial assignment therefore determine whether a nominally accurate estimate is actionable.

Current automation is strongest for immediate machine-level objectives, such as automatic discharge/loading and integrated product formation [12,13,14,15,16,17,18]. Leaf retention, nutrient preservation, fermentation, heating, and aerobic stability develop more slowly and are seldom available as real-time feedback. Quality-oriented control must therefore combine rapid proxy-state regulation with delayed measurements linked to the same material batch.

Previous reviews offer complementary views of forage mechanization. Machinery-centered syntheses describe equipment and mechanization development [1,2], production-stage reviews examine operations, such as drying [19], and precision-harvesting reviews connect yield and quality monitoring with site-specific management [3,4]. Other reviews compare sensing and data architectures [20,21] or address coordination among agricultural machines [22,23]. These perspectives describe equipment, measurements, or task coordination, but they seldom follow the same material from an observation through an available intervention to a delayed quality outcome. The present review uses control-relevant intermediate states to trace that continuity across hay, bale silage, and chopped silage production.

Actionability depends on whether an observation can be assigned to the affected material, interpreted before the intervention window closes, and converted into a decision that preserves downstream quality. Prediction accuracy and decision utility are distinct: an accurate estimate has little control value if it represents the wrong material portion, arrives after transport or processing delay, or lies outside the calibration domain.

The state-centered framework extends machinery- and sensor-centered syntheses in three ways. It uses control-relevant intermediate states to link mechanical action with downstream quality, interprets deviations through their propagation paths and remaining intervention time, and evaluates estimates through state validity, material alignment, decision utility, and downstream consequence. Rapid proxy states support immediate regulation, while batch-linked final-quality measurements provide delayed verification and model updating.

The review addresses four questions: how mechanical and environmental actions generate state changes and losses; which states can be recovered reliably during continuous operation; how aligned estimates can guide decisions and actuators while intervention remains possible; and how cross-stage records and final-product measurements can verify and update those decisions. Product-specific boundaries are retained for hay, bale silage, and chopped silage.

## 2. Review Scope, Literature Identification, and Evidence Appraisal

This critical narrative review synthesizes engineering evidence across the mechanized forage production chain, from machine action and material-state formation to sensing, decision or control, and downstream quality. Control-relevant intermediate states are used as the organizing unit because they connect machine settings and operating conditions with observable responses, available interventions, and delayed product outcomes. The analysis retains the distinct production and quality constraints of hay, bale silage, and chopped silage.

Contemporary literature was identified through searches of the Web of Science Core Collection and China National Knowledge Infrastructure (CNKI), with emphasis on publications from 2021 to 2026. Search concepts covered four linked areas: forage crops and products, mechanized harvesting and handling, sensing and state estimation, and decision support or machine control. Earlier publications were included when they established sensing principles, machine–material relationships, validated instrumentation, control concepts, or reference methods still relevant to current research. ISO and ASABE standards were consulted where they defined measurement, validation, or data-exchange requirements.

Evidence was interpreted according to its engineering relevance and applicability. Greater weight was placed on studies using established reference measurements, representative sampling, and independent validation under field or production conditions, while laboratory and bench studies were used mainly to establish mechanisms and technical feasibility. Reported R^2^, RMSE, SEE, SEP, bias, and accuracy values were interpreted within the material range, reference method, sampling design, calibration domain, and data partition of each study rather than compared directly across unlike experiments. Operational relevance was further judged by transferability, uncertainty, latency, material alignment, and whether the estimated state could support a feasible decision or actuation before the intervention window closed and could ultimately be linked to downstream forage or storage quality.

## 3. Quality Basis and Machine–Material–Environment Coupling in Mechanized Forage Production

Quality-oriented control requires explicit states that connect mechanical action with final forage quality. Cutting, compression, bending, impact, friction, shearing, and compaction act on material whose response changes with moisture, maturity, morphology, load, and environmental exposure. The resulting state becomes part of the operating boundary of the next operation.

### 3.1. Material Properties and Quality Development in Forage

Forage mechanics vary with species, maturity, organ, moisture, and post-cutting history. These properties govern machine load and the response to cutting, compression, impact, conveying, and compaction. Chen et al. [24] quantified alfalfa-stem mechanical and contact parameters relevant to cutting power, flow stability, and compression. High-moisture forage deforms and accumulates readily; as drying proceeds, stems and especially leaves become brittle, increasing fragmentation under repeated impact or friction.

Leaves generally contain more crude protein and less structural fiber than stems. Evidence on stem-fiber digestibility [25] and maturity-related metabolic change [26] links biomass accumulation and lignification to declining digestibility. Selective leaf loss therefore reduces both recovered mass and nutritive value, while harvest timing balances yield against mechanical fragility and feeding quality.

After cutting, respiration, moisture redistribution, oxidation, and microbial activity continue. Faster leaf drying can create a state in which brittle leaves coexist with wet stems. Conditioning and intensified drying may shorten the process [6,19], but excessive mechanical or thermal intensity can increase leaf shatter, effluent loss, heat damage, or energy demand. Control boundaries depend on the combined moisture, morphology, maturity, load, and environment.

Table 1 relates representative material states to observable indicators, dominant risks, candidate responses, and downstream verification. The responses are conditional on the current material and operating domain.

### 3.2. Quality-Loss Categories and Evaluation Metrics

Quality loss includes unrecovered material and deterioration in composition, nutritive value, hygienic quality, or storage stability. Four related categories are used here: quantitative, compositional, nutrient, and storage-quality loss. Across seven mower-conditioners, Shinners et al. [27] reported alfalfa leaf losses of 5.2–5.9% of dry matter; maturity and adjustment had greater effects than roll design, so the range is not a universal machine value.

Quantitative loss includes uncut forage, detached material, pickup or discharge loss, and transport or handling losses; cross-stage comparisons require a dry-matter basis. Compositional loss is especially important in legumes because selective detachment of nutrient-rich leaves lowers the leaf-to-stem ratio and increases the relative contribution of structural fiber.

Nutrient loss arises through respiration, rain leaching, sap or effluent loss, photo-oxidation, enzymatic degradation, and thermal damage. Harvest timing and initial moisture affect alfalfa-silage fermentation and microbial succession [28,29]. Across three harvests of low-dry-matter forage, Randby and Bakken [30] reported total dry-matter losses of approximately 17% for bunker silage and 7% for round-bale silage, illustrating the pathway-specific interaction among recovery, fermentation, and aerobic stability.

Storage-quality loss may appear hours or weeks after field operations. Non-uniform moisture, inadequate compaction, delayed wrapping, film damage, or incomplete sealing can promote heating, mold, undesirable fermentation, and aerobic deterioration. Final failure can therefore be a delayed expression of an earlier state deviation.

Because different mechanisms can produce similar end symptoms, diagnosis requires indicator combinations. Table 2 links abnormal patterns with plausible stages, discriminating evidence, confounders, and control implications; it is a traceability aid rather than a deterministic causal lookup.

Evaluation connects initial state, operational performance, intermediate response, final nutritive quality, and storage safety. Initial descriptors define the validity domain; operational variables characterize stubble, conditioning, geometry, pickup, particle formation, flow stability, and bale density; delayed measurements determine whether these states preserved the intended product.

Nutritive assessment commonly includes crude protein, fiber fractions, starch, water-soluble carbohydrates, digestibility, and energy indices. Hay additionally requires final moisture, uniformity, heating, mold, and density; silage requires pH, fermentation acids, ammonia nitrogen, dry-matter recovery, and aerobic stability. No single index represents recovery, nutritive value, processing quality, and storage risk.

Reporting conventions also affect interpretation. Moisture must be identified on a wet or dry basis, while losses and composition are generally compared on a dry-matter basis. Sampling position and timing are critical for windrows, chopped material, and bales; means for moisture, particle length, linear density, or bale density are most useful when accompanied by dispersion where heterogeneity affects risk.

Metrics have different temporal roles. Load, flow, moisture, and product-formation states can support immediate adjustment. Leaf retention, nutritive composition, fermentation, heating, and aerobic stability arrive too late for rapid control but can verify proxy states, operating boundaries, and batch-level outcomes.

### 3.3. Cross-Stage State Propagation and Product-Specific Boundaries

Forage response emerges from machine–material–environment coupling. Geometry, clearance, speed, pressure, and load act on material that varies in species, maturity, moisture, canopy structure, morphology, and mechanics, while weather and soil conditions alter drying, contamination, handling, and the available operating window.

A fixed setting does not produce a fixed response. Wet forage resists brittle fracture but is prone to wrapping, blockage, rebound, and incomplete compaction; drier forage may flow more readily while leaf fragmentation increases. Stem diameter, maturity, terrain, and weather further shift the admissible balance among disturbance, speed, and compaction. Settings are valid only within a defined operating domain.

Material states propagate across operations. Front-end geometry, tissue damage, and drying conditions become downstream variation in feed rate, particle structure, compaction, and storage environment. The output state of one operation becomes part of the operating boundary of the next.

Moisture distribution, leaf integrity, windrow geometry and linear density, mass flow, particle state, bale density, and pore structure are the principal intermediate links between mechanical action and delayed quality [3]. They reveal how a disturbance propagates and where corrective action remains possible.

**Figure 1:**
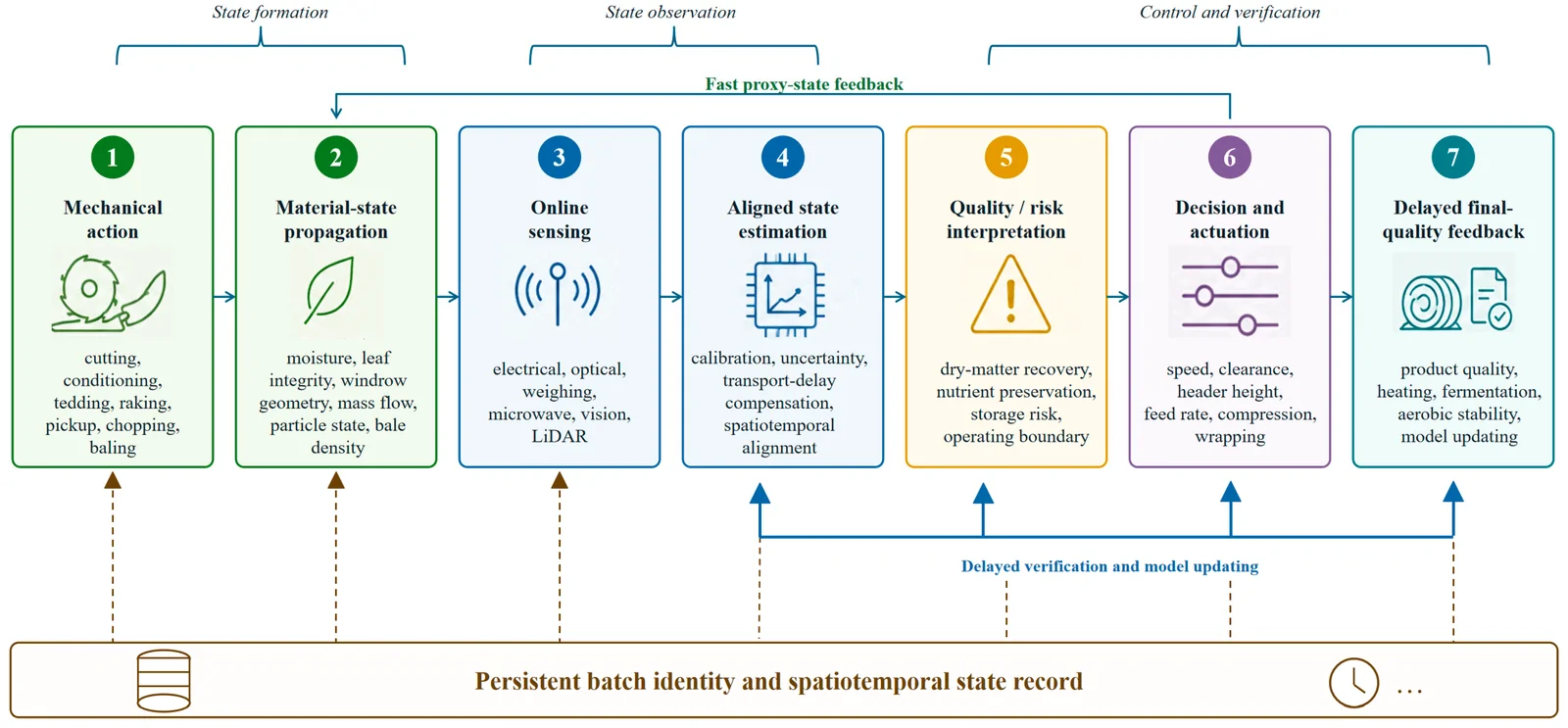
Quality-oriented analytical framework for mechanized forage production. Mechanical action and material-state propagation define state formation; online sensing and aligned state estimation provide state observation; quality/risk interpretation and decision and actuation support control. Fast proxy-state feedback enables immediate regulation, whereas batch-linked final-product and storage measurements provide delayed verification and model updating. Persistent batch identity and spatiotemporal state records maintain traceability across the production chain.

Figure 1 organizes this state-centered analysis into state formation, state observation, and control and verification. Mechanical action generates states that propagate across operations; online measurements are converted into aligned estimates for risk interpretation and operational decisions. Persistent batch identity and spatiotemporal records link downstream outcomes with upstream material and machine states.

The fast loop regulates operations from online proxy states. The delayed loop associates final-product and storage outcomes with the same batch to verify and update models or operating boundaries. Figure 1 is an analytical framework, not a prescribed controller architecture.

Control boundaries also depend on product pathway. Hay, bale silage, and chopped silage share measurable states but differ in decision timing, admissible moisture, compaction requirements, and dominant safety risks. Table 3 summarizes the corresponding decisions and non-negotiable boundaries.

The same moisture, flow, or geometry estimate may therefore justify different actions. Interpretation requires the intended product and its current operating boundary.

Product-specific boundaries also determine what can be corrected downstream. A hay windrow that remains too wet may still be redistributed or delayed, whereas excess moisture detected after baling may require segregation and storage monitoring. For bale silage, density or film defects remain correctable only until wrapping is complete; for chopped silage, particle and flow deviations become difficult to reverse after packing. State interpretation therefore includes both the target pathway and the latest stage at which compensation remains physically possible.

## 4. Quality-Loss Formation and Material-State Evolution Across Mechanized Forage Operations

This section follows the production chain from mechanical action to state change, downstream consequence, and the remaining opportunity for intervention.

**Figure 2:**
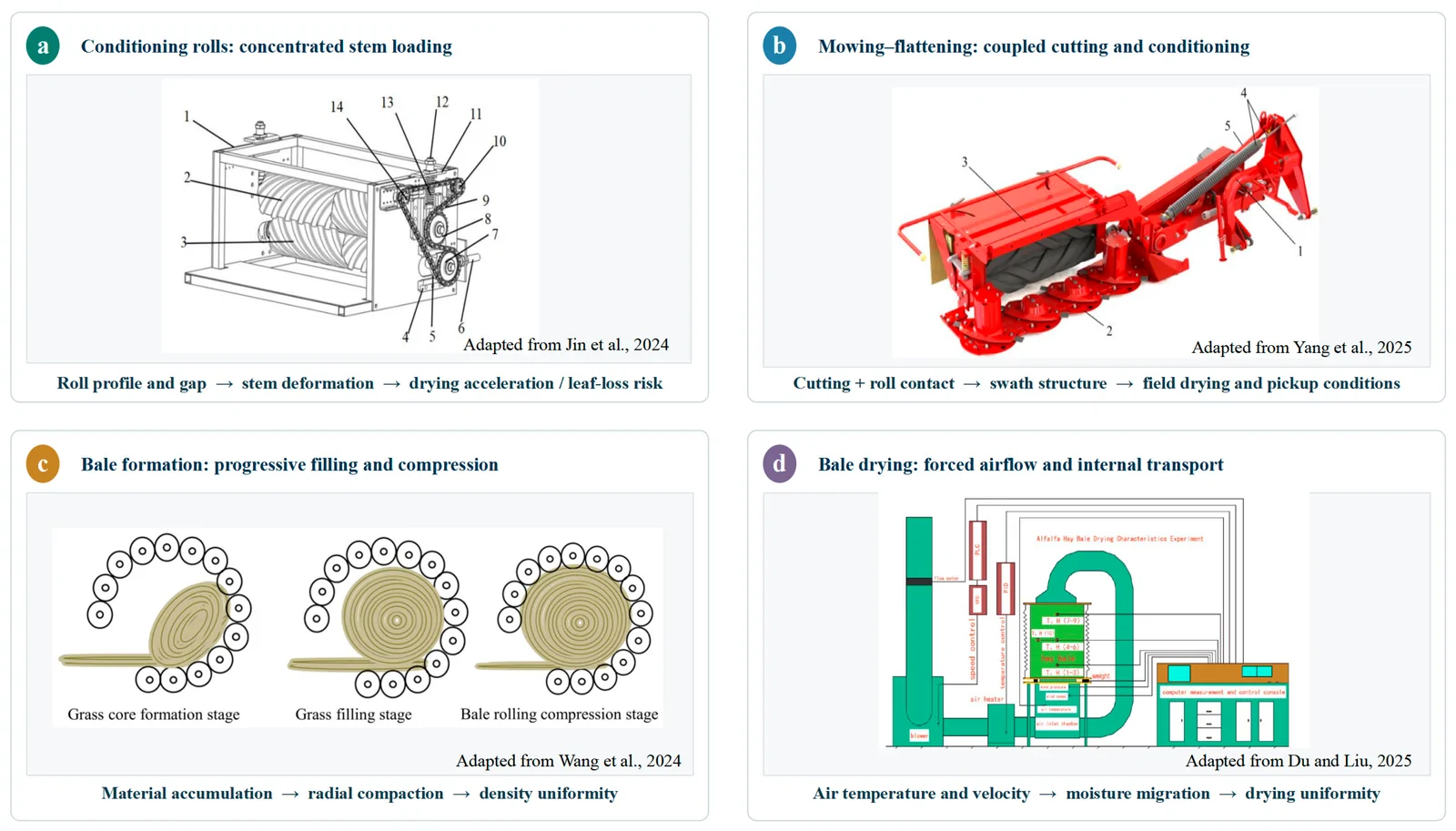
Representative machine–material interactions, intermediate material states, and downstream quality-loss pathways across mechanized forage operations. Panels: (**a**) 1, body frame; 2, upper conditioning roller; 3, lower conditioning roller; 4, guide rail; 5, sliding bearings; 6, horizontal guide screw; 7, lower sprocket; 8, upper sprocket; 9, chain; 10, tensioner pulley; 11, retainer plate; 12, vertical guide screw; 13, mechanical spring; and 14, tensioner pulley. (**b**) 1, suspension frame; 2, mowing device; 3, flattening and modulating device; 4, hydraulic lifting system; and 5, contouring mechanism. Adapted from Refs. [31,32,33,34]. Under the Creative Commons Attribution 4.0 license.

Figure 2 provides a stage-level overview of how machine–material interaction modifies intermediate states and changes drying, loss, compaction, or storage risk.

Mechanical intensity is not an objective in itself; the produced state determines both downstream risk and the remaining intervention window.

### 4.1. Mowing and Swath Formation

Mowing initiates post-cutting evolution and determines recoverable yield and the material form entering conditioning. Cutting height, cutter condition, speed, and travel rate affect removed biomass, composition, contamination, stubble uniformity, and transient flow [35]. Lower stubble can recover more biomass but include coarser basal stems and greater contamination risk; excessive height leaves harvestable material. These outputs define the first downstream boundary.

Insufficient cutting velocity can push or repeatedly cut stems, whereas excessive speed raises power demand and may scatter material. The control objective is a uniform, recoverable swath rather than maximum cutting intensity.

Swath width, thickness, and uniformity govern radiation and airflow exposure [19]. Thick or narrow regions retain moisture and amplify gradients, while excessive spreading increases the need for later consolidation. These geometry states determine early drying and the workload transferred to tedding or raking.

### 4.2. Mechanical Conditioning

Conditioning reduces stem moisture-transfer resistance by applying compression, bending, crushing, impact, or abrasion [27,36,37]. Roll systems can limit fragmentation relative to finger or flail systems, but response varies with species, stem diameter, maturity, moisture, mat thickness, and throughput.

Multi-objective experiments show that conditioning degree, material loss, and specific energy can be balanced through roll profile, speed, clearance, and feed rate [36,37]. Reported optima are strongly condition-dependent and should be interpreted through stem damage, swath geometry, moisture distribution, and drying response rather than transferred directly between machines or crops.

Uniform treatment accelerates drying by increasing tissue permeability. Local accumulation leaves wet internal stems and brittle exposed leaves, whereas excessive intensity increases leaf or effluent loss. The useful output is a sufficiently uniform moisture-transfer state, not maximum visible stem damage.

### 4.3. Tedding and Raking

Tedding redistributes forage for drying, while raking consolidates it for pickup. Both impose repeated impact, bending, friction, and tine contact, so moisture uniformity, leaf retention, contamination, and windrow structure must be considered together [38,39].

Tedding benefit declines as leaves become brittle. Seasonal experiments found that drying response varied with harvest period and weather and that an additional pass could provide little moisture reduction while increasing leaf loss [38]. Moisture decline and stem–leaf divergence determine whether another pass remains within an effective intervention window.

Raking must create a pickup-compatible windrow without excessive tine–ground interaction or material disturbance. In one commercial alfalfa field, cumulative dry-matter loss across mowing, raking, and baling was 30.93%, with 17.35% attributed to raking; changing mowing–raking direction reduced total loss by 2.29 percentage points [39]. The field-specific result identifies raking as a critical transition rather than a universal loss fraction.

The state entering pickup is defined jointly by moisture distribution, leaf integrity, ash contamination, windrow width, and linear-density uniformity. An acceptable mean moisture can conceal brittle leaves, wet stems, or an irregular windrow.

### 4.4. Pickup, Feeding, and Chopping

Pickup and feeding convert spatial windrow variability into temporal feed-rate variation. Pickup height, tine speed, travel speed, windrow linear density, and moisture influence residue, soil intake, impact damage, and transfer stability [39]. Resulting load fluctuations propagate into chopping, conveying, or baling as variable particle size, overload, blockage, or density non-uniformity [40,41].

Chopping changes particle length, size distribution, and surface area. Actual performance reflects theoretical length of cut, material orientation, moisture, knife condition, and knife–shearbar clearance. Chen et al. [40] detected contact from vibration and maintained clearance automatically, linking an observable machine state to direct correction of particle formation and energy loss.

Processing intensity also affects feeding value. Experimental comparisons show that processor clearance and theoretical length of cut interact in their effects on starch digestion, physically effective fiber, and milk-production responses [41]. Settings therefore remain conditional on maturity, moisture, compaction, and feeding objectives.

For chopped silage, feed stability and particle state also constrain pneumatic conveying. Insufficient airflow causes accumulation; excessive airflow increases fine-particle loss. Feed rate, particle distribution, processing degree, fragment loss, and discharge continuity form the relevant state set.

### 4.5. Baling and Wrapping

Baling converts a heterogeneous stream into a discrete product whose density, geometry, pore structure, and moisture distribution govern handling and storage [42,43,44]. Uneven feeding creates local compression differences and gradients that cannot be inferred from mean chamber pressure alone.

Compaction reduces transport and storage volume, but its benefit depends on moisture, particle structure, and product pathway. Tests with grass–legume material confirmed that density responds jointly to moisture, particle length, and pressure [42]. Dense wet hay may retain heat, whereas bale silage requires sufficiently high and uniform density for air exclusion; excessive moisture or uneven compression can cause effluent, deformation, or film stress.

Finished-bale state reflects pickup, filling, compression, tying or wrapping, and discharge. Uniform feeding and pre-compression improve density distribution [43], while integrated pickup–baling–wrapping reduces transfers and repeated handling [13]. Packaging reliability is part of product formation rather than a separate endpoint.

For wrapped silage, irregular geometry, protruding stems, unstable tension, or inadequate overlap permit oxygen ingress. In alfalfa-dominant bales, film treatments had limited overall effects on major quality traits, but surface fermentation differed and oxygen-barrier film remained vulnerable to coarse stems [44]. Geometry, density distribution, film integrity, and sealing jointly define storage entry.

### 4.6. Drying and Storage

Drying and storage convert earlier mechanical effects into biochemical and hygienic outcomes. Field curing uses little dedicated energy but increases weather exposure; heated-air drying reduces exposure while trading energy demand against color and biochemical preservation [6]. Relevant outcomes are processing time, moisture uniformity, nutrient retention, and energy demand.

In baled forage, pore structure, initial moisture, density, dimensions, airflow, and air temperature govern heat and mass transfer. Hot-air experiments found surface–core and windward–leeward gradients [34]. Higher temperature or velocity accelerates drying but raises energy use, while lower density improves penetration at the expense of transport efficiency. The target is safe and sufficiently uniform final moisture.

Silage requires rapid anaerobiosis through suitable incoming moisture, particle length, packing density, and sealing. Greater density can suppress aerobic deterioration in wilted alfalfa [45], while storage temperature affects nutrient loss, microbial succession, and fermentation products [46]. These outcomes inherit the combined harvesting, chopping, compaction, and sealing state.

### 4.7. Cross-Stage Propagation of Quality Losses and Material-State Trade-Offs

Quality loss is cumulative and path-dependent. Mowing and conditioning establish geometry, tissue damage, and drying conditions; tedding and raking reshape moisture and windrow distributions; pickup converts spatial variation into feed-rate variation; chopping changes particle structure; and baling or ensiling converts these states into density, gas transfer, fermentation, and storage conditions.

The recurring trade-offs are biomass recovery versus contamination, drying rate versus leaf retention, reduced particle size versus power and physically effective fiber, and compaction versus heat dissipation, effluent, or energy use. Their balance shifts with the current state and product pathway.

Representative pathways make this propagation explicit. Non-uniform moisture may create unequal deformation, compaction, and air exclusion; an acceptable mean can still conceal local wet zones associated with heating, mold, undesirable fermentation, or reduced storage stability [28,29,30,45,46]. Once leaves become brittle, excessive tedding or raking disturbance may increase selective leaf fragmentation and loss, lowering the leaf-to-stem ratio and removing a nutrient-rich fraction relative to structural fiber [27,38,39]. Uneven windrow linear density can become temporal feed-rate fluctuation at pickup, destabilizing chopping or bale formation and increasing particle-size or density variability, with possible downstream effects on compaction and storage uniformity [39,40,41,42,43,47]. The severity and direction of these outcomes remain conditional on material, machine, weather, and product pathway. Thus, intermediate states are not merely sensing targets; they are propagation variables linking mechanical action with delayed quality consequences.

Control relevance follows a four-part sequence: state deviation, downstream propagation, remaining intervention window, and irreversible quality consequence. Table 4 identifies observable states and the latest practical compensation point before each abnormality becomes embedded downstream.

Deviations are easiest to correct near their origin. Once embedded in particle structure, bale architecture, or storage conditions, the intervention window narrows or closes. Online sensing becomes control-relevant only when detection precedes that loss of corrective opportunity.

## 5. Online Sensing and Estimation of Control-Relevant Forage States

Regulation requires state observations that are sufficiently representative, timely, and robust while corrective action remains possible. Wang et al. [48] integrated mass flow, travel speed, rotational speed, torque, pressure, and temperature on a forage harvester, moving from isolated signals toward joint material and process observation. Control readiness additionally depends on calibration transfer and drift, uncertainty, latency, spatiotemporal alignment, operating-domain validity, and continuous-deployment reliability.

**Figure 3:**
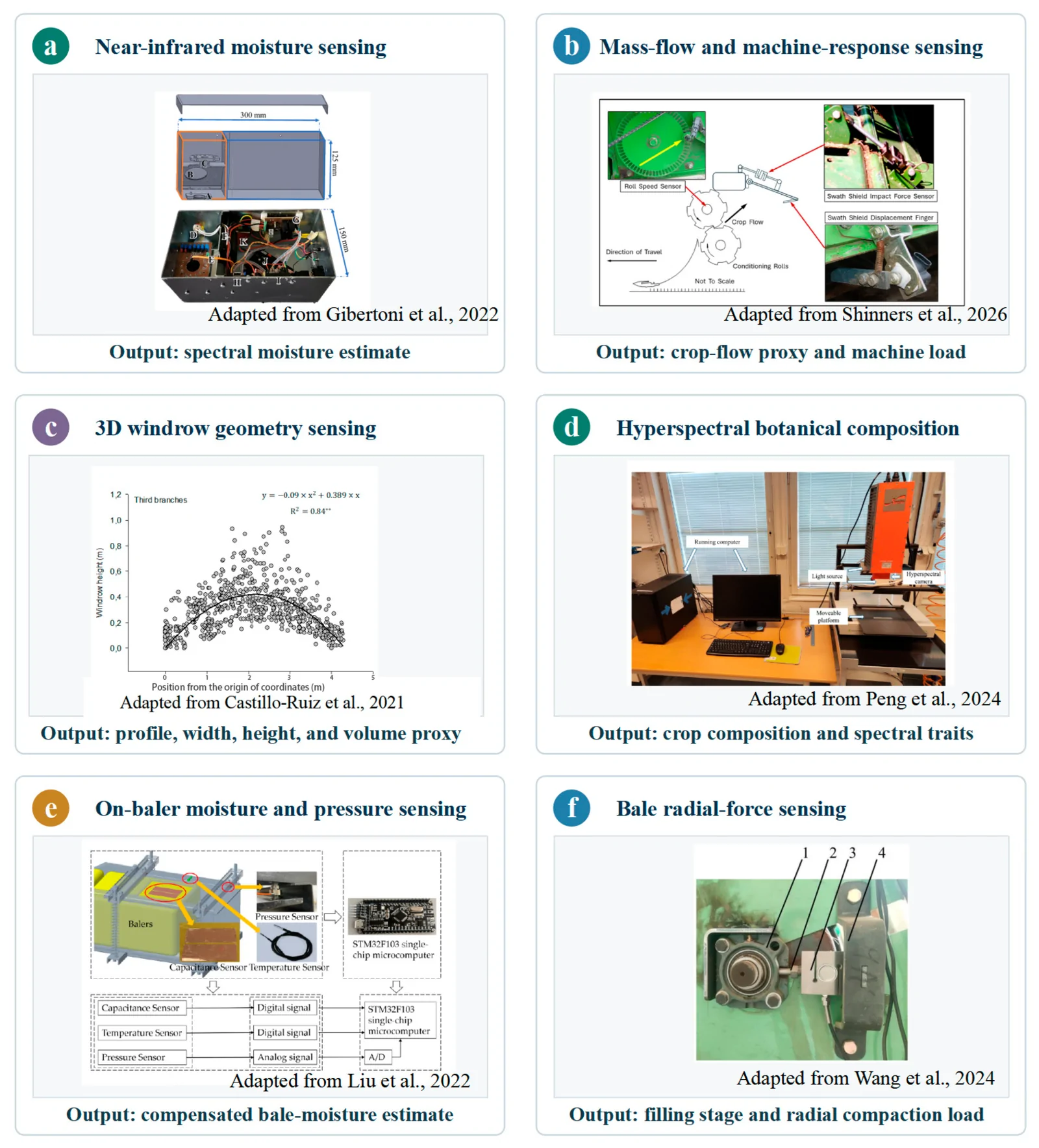
Representative sensing configurations for control-relevant forage and machine states. Panels: (**a**) A, optical-window mount; B, lamp mount; C, optical-fiber head holder; D, halogen lamp; E, OH-silica optical fiber; F, cooling fan; G, control board; H, microcontroller-unit board; I, conditioning board; J, near-infrared spectrometer; and K, DC–DC converter. (**c**) open circles are 3D time-of-flight measurements, the solid curve is the polynomial fit, y is windrow height, R^2^ is the coefficient of determination, and ** indicates *p* < 0.01. (**d**) Leader lines identify the running computer, light source, movable platform, and hyperspectral camera. (**e**) Arrows indicate signal flow from the capacitance, temperature, and pressure sensors to the microcontroller. (**f**) Leader lines identify the instrumented bearing and sensor locations. 1. Bearing seat; 2. Measurement column; 3. S-type tensile pressure sensor; 4. Sensor Support. Adapted from Refs. [11,33,47,49,50,51]. Under the Creative Commons Attribution 4.0 license.

Figure 3 shows how spectral, geometric, and interaction-based measurements recover complementary state components. Panel (c) illustrates optical windrow-profile reconstruction in adjacent biomass machinery; forage-specific performance remains to be tested across materials and field conditions.

No single modality reconstructs the complete forage state. Spectra and images observe composition or external structure, whereas force, pressure, displacement, and weighing describe machine response. Fusion is justified when complementary signals resolve a decision-relevant ambiguity within the available response time and intervention window.

### 5.1. Moisture Content and Drying-State Sensing

Moisture governs flexibility, drying, mechanical damage, compaction, and storage stability. For control, it describes both the evolving field state and the condition entering product formation or storage.

Online moisture measurement mainly uses electrical resistance, capacitance, microwave response, and NIR spectroscopy. Electrical systems integrate readily with machinery but remain confounded by density, temperature, contact pressure, stem–leaf distribution, and orientation. Yang et al. [52] combined mass change, airflow, temperature, and material position with a convolutional neural network, illustrating how auxiliary signals can separate water variation from process effects.

A reliable estimate requires an explicit wet- or dry-basis reference, sampling position, oven method, and temperature treatment. ANSI/ASAE S358.3 defines procedures for different forage forms and warns that drying above 65 °C may be unsuitable before some chemical analyses [53]. Independent-sample bias, standard error, repeatability, density sensitivity, and contact effects are needed alongside calibration coefficients.

NIR estimates moisture from water- and organic-bond absorption, but water also interferes with other predictions. Yang et al. [54] reported reduced nutritive-calibration accuracy in fresh forage because of absorption and irregular scattering. For freshly harvested alfalfa, Gibertoni et al. [49] obtained R^2^ = 0.985 and an estimated relative RMSE of 7.1% with a portable 900–1700 nm system. The 12-sample dataset establishes feasibility, not continuous-field robustness.

Short-wave infrared (SWIR) hyperspectral imaging adds spatial information. Chu et al. [55] identified informative bands near 970 and 1450 nm across alfalfa product forms, supporting reduced-band sensors. Transfer remains sensitive to product-dependent pathlength and scattering, illumination, surface relief, dust, and vibration; the calibration population must represent presentation as well as moisture.

For online use, moisture is better treated as a spatially and temporally varying state than as a single sensor reading. A representative estimate may require several contact points, repeated spectra, or fusion with windrow geometry and process load. The estimate must also carry its basis, calibration status, uncertainty, and acquisition location so that subsequent control does not combine incompatible moisture definitions.

### 5.2. Mass-Flow, Yield, and Feeding-State Sensing

Mass flow links productivity, load, yield mapping, and capacity regulation, but estimation is confounded by compressibility, heterogeneity, and moisture.

Indirect sensing uses feed-roll displacement, torque, hydraulic pressure, impact force, capacitance, or dynamic weighing. In wilted forage at 45–78% moisture and 1–15 kg s^−1^, Savoie et al. [56] reported R^2^ = 0.951 for spout impact force and R^2^ = 0.863 for feed-roll displacement; combining impact force with capacitance reduced SEE from 0.7 to 0.3 kg s^−1^. Fusion separates coupled material effects rather than merely increasing channel count [4].

Machine networks can synchronize material and load variables. Wang et al. [48] related feed rate to loads across header, chopping, and discharge units. In a pull-type harvester, Mohsenimanesh et al. [57] obtained R^2^ = 0.97 between feed-roll opening and mass flow at a 15 mm theoretical length of cut, but R^2^ = 0.59 at 9.5 mm, showing that chopping configuration changes the measurement relationship.

Dry-matter yield estimation also requires moisture conversion and correct spatial assignment. Global Navigation Satellite System (GNSS), travel speed, and moisture support this step, but material transport between pickup and sensor introduces a delay that must be compensated before measurements are mapped or used for control.

Shinners et al. [47] estimated alfalfa flow during mowing from conditioner-roll speed, windrow-shield impact, and tine displacement. The roll-speed model achieved R^2^ = 0.89 and RMSE = 3.4 kg s^−1^ over 4–55 kg s^−1^; independent validation yielded R^2^ = 0.87 and RMSE = 2.62 kg s^−1^. Control assessment therefore includes independent-condition transfer, moisture compensation, delay correction, and spatial fidelity, with georeferenced records aligned where appropriate with ASABE S611 [58].

Mass-flow uncertainty affects more than yield maps. It changes the inferred specific energy, the feedforward demand presented to chopping or baling, and the dry-matter basis used for loss accounting. A useful estimator therefore reports dynamic response and bias across flow transitions, not only an aggregate fit obtained after steady-state averaging.

### 5.3. Windrow Geometry and Operating-Scene Sensing

Windrow centerline, width, height, cross-sectional area, and discontinuity determine pickup trajectory and temporal inflow. Red–green–blue (RGB) or depth cameras and LiDAR observe this upstream geometry before machine interaction. RGB provides low-cost texture and color information but is sensitive to illumination, shadow, dust, and weak crop–soil contrast; LiDAR is less illumination-dependent and directly recovers three-dimensional structure.

Castillo-Ruiz et al. [50] reconstructed cross-sectional geometry and bulk-volume proxies in pruning-residue windrows with a 3D time-of-flight camera. Pindl et al. [59] used filtering and principal-component analysis to estimate windrow position and orientation from vehicle-mounted LiDAR and fused successive frames into a field-level map.

Scene sensing also supports chopped-forage discharge. Liu et al. [14] identified trailer boundaries and pile contours, estimated angle of repose and available volume, and selected the desired impact location. The decision variable is material distribution and remaining capacity, not object detection alone.

Geometry does not equal mass because bulk density varies with moisture, composition, particle dimensions, and compression history. Volume-based quantity estimates therefore need complementary moisture, pressure, or weight information. Dynamic use also requires processing latency, localization error, occlusion recovery, and alignment among sensing, material motion, and actuation to be reported.

### 5.4. Sensing of Nutritive Quality and Material Composition

Laboratory analysis remains the compositional reference but cannot represent heterogeneous moving material continuously. Spectroscopy can estimate crude protein, fiber fractions, digestibility, botanical composition, and related attributes during or shortly after harvest.

Transfer from laboratory calibration to machine-presented forage is the main constraint. Cultivar, maturity, harvest cycle, region, moisture, scattering, and presentation shift spectral distributions [54]. Models trained on dried, ground material require independent validation on fresh, unground material in motion, with standardization, calibration transfer, updating, and out-of-domain detection used to define applicability.

Miniaturization reduces instrument size and cost but not sampling error. Melendreras et al. [60] increased sampling area and repeated scans of heterogeneous alfalfa. With 111 undried farm samples, Yamada et al. [8] found translation or rotation more robust than static scanning, while moisture still impaired lignin- and digestibility-related predictions. Representative presentation and optical-path control remain essential.

Hyperspectral imaging adds spatial heterogeneity. Peng et al. [51] estimated legume proportion in mixed forage, and Jeong et al. [61] obtained calibration R^2^ = 0.94 for leaf and stem proportions in controlled alfalfa-hay mixtures. Such estimates may indicate selective leaf loss but require continuous-flow validation under overlap, occlusion, variable packing, and changing moisture.

Particle and kernel processing are less mature sensing targets. Van Puyenbroeck et al. [62] used hyperspectral classification to distinguish kernels from non-kernel material and reduced the band set for computation. Rocha et al. [63] acquired high-speed images of moving corn silage and estimated kernel processing score against sieving (r(38) = 0.98). Extension to general forage particle distributions requires standardized reference sizing, representative sampling, and latency testing.

Controlled studies continue to show strong spectral potential. Benelli et al. [64] obtained a prediction coefficient of determination (R^2^p) of 0.96 and a root mean square error of prediction (RMSEP) of 2.95 g per 100 g fresh weight for dry matter from 324 samples. With 348 spectra of fresh, unground alfalfa silage, Wu et al. [65] reported cross-validated R^2^ values of 0.986 for dry matter, 0.943 for water-soluble carbohydrates, and 0.958 for lactic acid. Neither result alone establishes continuous-flow performance under vibration, dust, and presentation change.

Calibration maintenance remains necessary after deployment. Shinners et al. [66] found systematic differences between on-site near-infrared spectroscopy (NIRS) and commercial laboratory analysis of fresh maize. For two handheld instruments calibrated with 317 dried and ground grass–legume samples, Castillo et al. [67] reported r^2^ ≥ 0.90 for crude protein and in vitro organic-matter digestibility, bias from −0.22% to −0.17%, and SEP of 1.8–2.3%. Operational evaluation must separate repeatability, prediction error, transferability, and decision utility.

Across spectroscopy studies, high within-dataset performance is repeatedly easier to obtain than transfer across instruments, seasons, crop forms, and reference laboratories. Independent batches should preserve the deployment structure: spectra from the same field, bale, or harvest event cannot be divided across calibration and test sets when the intended claim is batch-level transfer. Reporting the validity domain is part of the result rather than a secondary limitation.

### 5.5. Sensing of Bale and Product-Formation States

Baling creates a discrete unit whose mass, density, moisture, geometry, and surface integrity affect transport and storage. Measurements during formation or discharge can also establish a traceable bale-level endpoint.

Mass and density can be inferred from dynamic weighing, hydraulic pressure, chamber force, and dimensions. Force and pressure observe compression before completion; discharge weighing provides a finished-product estimate.

Wang et al. [68] related radial force and changing chamber geometry to density in large steel-roller round balers and reported 93–97% prediction accuracy. The approach observes mean compaction online but does not reveal internal heterogeneity; the reported performance was obtained for the tested material and configuration.

Uneven transverse feeding and moisture gradients create local density variation that can promote heating or mold despite acceptable mean density. Mass and mean density must therefore be interpreted with moisture, geometry, and uniformity; wrapped silage additionally requires film-overlap, deformation, and sealing indicators.

Cassanelli et al. [69] developed a 2.44 GHz microwave attenuation system for rapid whole-bale moisture measurement in straw. Application to forage requires separation of moisture, density, dimensions, and dielectric properties and evidence that localized wet zones are detected rather than averaged.

Bale sensing links process monitoring with product verification, but internal moisture distribution, pore structure, and later storage behavior remain latent states requiring proxy estimation and downstream validation.

### 5.6. Multi-Sensor Fusion, Calibration, and On-Machine Integration

No sensing principle uniquely identifies forage state. Electrical response combines moisture, density, temperature, and contact; force combines material interaction with machine dynamics; vision resolves external geometry but not internal moisture; and spectra depend on composition and presentation. Any sensing combination must remain within sampling-rate, communication, power, and processing constraints [20,21].

Sensor fusion is most useful when a second modality separates a confounder that matters to the control decision. Temperature, density, and contact pressure can distort electrical moisture response; temperature or load measurements can help distinguish those effects from water content. Camera or LiDAR data describe windrow geometry, but feed-rate control also needs information on moisture, bulk density, or machine load because volume alone does not determine mass. Spectral estimates likewise vary with illumination, dust, surface relief, packing, and material position, and may benefit from imaging or machine-state data. Additional channels are warranted only when they reduce uncertainty enough to improve the state estimate or decision without excessive latency, calibration, computation, communication, or maintenance. Fusion should be judged by the ambiguity it resolves, not by the number of sensors.

State estimation is distinct from sensor aggregation. It requires coordinate transformation, temporal alignment, transport-delay compensation, calibration-state checks, and uncertainty propagation. Directly unobservable properties, such as leaf integrity, internal moisture distribution, or pore structure are latent states inferred from validated proxies. A usable estimate records the value, time, location, material or batch identity, uncertainty, and validity status [20,21,70].

Table 5 summarizes reported sensing performance alongside the deployment conditions relevant to continuous use.

Measurements acquired at different rates, positions, fields of view, and coordinate systems must be assigned to the same material. Effective delay includes acquisition, computation, communication, material transport, and actuator response. Filtering and calibration-state management are therefore part of estimation rather than post-processing details.

Operational models also require out-of-domain detection to prevent apparently precise predictions for unfamiliar material. Band reduction, model compression, and edge inference can reduce latency [70], while a validity flag allows control logic to enter a defined fallback state when confidence is inadequate.

Continuous measurement is feasible for moisture, mass flow, geometry, selected constituents, processing indicators, and mean bale density. Leaf integrity and internal pore structure remain mainly proxy-derived, and much evidence comes from laboratory tests, small datasets, or random partitions. ISO 12099 [71], ASABE S631 [72], and forage-NIR reporting guidance [73] emphasize documented references, calibration maintenance, bias, SEP, slope, intercept, and independent validation.

Long-term calibration validity must also be tested under persistent dust, vibration, optical-window contamination, temperature variation, crop-residue accumulation, component wear, and seasonal shifts in material presentation, because short trials may not reveal the resulting drift, recalibration demand, maintenance burden, or recovery time.

Prediction metrics from controlled experiments, small samples, or random data partitions do not by themselves establish readiness for continuous field operation. Operational evidence also needs representative sampling and stable calibration under changing conditions. Validation across independent seasons, sites, machines, batches, and operating conditions is more informative than another random split, especially when uncertainty and out-of-domain behavior are reported explicitly.

Timing and alignment are equally important. An estimate must remain valid and assigned to the affected material as it moves from the sensing point to the actuator. End-to-end tests can combine synchronized reference samples and machine records to quantify sensing-to-actuation delay and alignment error; laboratory averages cannot reveal whether the sensor and actuator acted on different material.

Deployment robustness also depends on how the system responds to contamination, missing data, calibration failure, or inputs outside the model domain. The estimator may hold the last valid value briefly, revert to a conservative setting, request operator confirmation, or suspend quality-dependent adjustment while safety control continues. Reporting availability, recovery, and fallback performance shows whether sensor fusion improves reliability. The final criterion is whether the estimate improves a defined operational decision.

## 6. Sensing-Driven Decision-Making and Intelligent Control of Forage Machinery

Intelligent control begins when an aligned state estimate changes a decision and actuator command while intervention remains possible. A complete route links observed state, decision variable, actuation, immediate response verification, and a downstream quality constraint. Safety and overload require rapid regulation; moisture transition, leaf retention, particle state, bale density, and storage risk impose slower limits on admissible settings.

**Figure 4:**
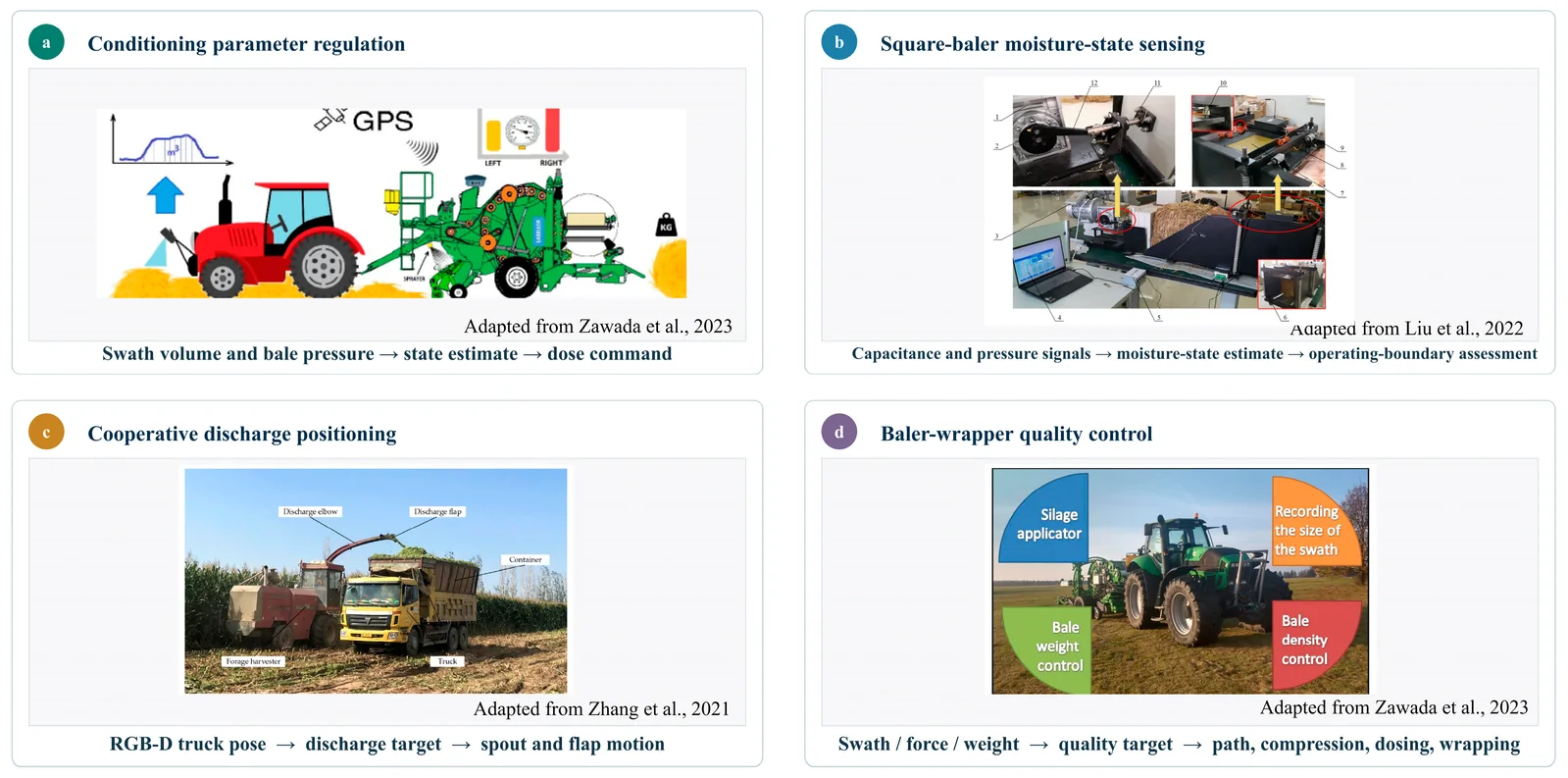
Representative sensing-to-actuation routes in forage machinery. Panels: (**b**) 1, reducer; 2, crank wheel; 3, motor; 4, monitoring software; 5, moisture-content device; 6, rear view of the press-baling chamber; 7, backplane of the capacitor plates; 8, hot-rolled steel groove; 9, limit wheel and support frame; 10, pressure sensor; 11, piston push rod; and 12, connecting rod. (**c**) Labels identify the forage harvester, discharge elbow, discharge flap, truck, and container. (**d**) The colored sectors identify the silage applicator, swath-size recording, bale-density control, and bale-weight control. Adapted from Refs. [11,15,16]. Under the Creative Commons Attribution 4.0 license.

Figure 4 illustrates representative sensing-to-decision and sensing-to-actuation routes in forage machinery.

The examples show rapid regulation of load, material flow, positioning, and product formation. Nutritive quality, fermentation, heating, and aerobic stability require delayed batch-linked verification.

Evidence is most mature for actuator position, load, clearance, trajectory, and selected product-formation tasks. Moisture, geometry, composition, and particle sensing often inform decisions without closing a quality-constrained loop, and few production studies validate the complete sensing–decision–quality chain.

Multivariable control studies in adjacent harvesting machinery provide transferable methodological evidence for coupled-state regulation, although they do not constitute direct validation of forage-quality control [74].

### 6.1. Harvest-Timing and Operational-Planning Decisions Under Quality Objectives

Harvest timing defines the initial state of the mechanized chain. Biomass often increases with maturity while leaf-to-stem ratio, crude protein, and fiber digestibility decline; rainfall, humidity, radiation, wind, and the rain-free period determine whether the intended sequence can be completed. The decision is product- and capacity-dependent rather than calendar-based.

Decision support combines crop, weather, and spatial information. Li et al. [5] linked alfalfa quality and meteorology to hay-harvest suitability, while remote-sensing studies [75,76] characterized yield and quality variation. Yu et al. [77] estimated 16 traits and reported R^2^ = 0.921 and RMSE = 300 kg ha^−1^ for dry-matter yield; Fu et al. [78] reported R^2^ = 0.92–0.95 for several quality traits. Field prioritization remains contingent on transfer across years, regions, crop states, and decision contexts.

Product pathway changes the boundary. Hay needs sufficient curing time without excessive brittleness; bale silage accepts shorter field drying but requires suitable fermentation moisture before wrapping; chopped silage emphasizes maturity, processability, particle state, and compaction. Planning also aligns field sequence with machine capacity so that mowing does not outpace collection and extend exposure.

Planning must also respect the intervention window of each field. A high-quality stand should not be mown simply because its estimated maturity is favorable if available tedding, transport, or baling capacity cannot complete the pathway before rain or excessive field drying. Decision support therefore couples field priority with executable downstream capacity.

### 6.2. State-Dependent Control of Mowing, Conditioning, Tedding, and Raking

Front-end control selects cutting height, travel speed, conditioning intensity, swath formation, tedding, and raking from crop geometry, moisture, load, ground condition, weather, and the remaining intervention window.

For mowing, ground profile, canopy height, lodging, and load support cutting-height and speed adjustment. Conditioning intensity is then constrained by stem diameter, moisture, mat thickness, and available drying time. Roll experiments show that high conditioning can be achieved without extensive fracture when structure and settings are matched [36]; dry or fragile material requires lower compression and impact.

Moisture decline and stem–leaf divergence determine whether another tedding pass remains beneficial and, if so, whether rotor or travel speed should be reduced [38]. Raking uses windrow position and three-dimensional structure [59] to create a pickup-compatible state with acceptable width, linear density, moisture, and uniformity. These outputs remain linked to the relevant field area or batch for downstream use.

Immediate verification differs by operation. Mowing control can verify stubble and load response; conditioning can verify pressure, roll separation, or a damage proxy; tedding and raking can verify geometry, moisture redistribution, and loss indicators. These fast responses confirm actuator execution, while later drying and leaf-retention measurements determine whether the intervention remained beneficial.

### 6.3. State-Dependent Control of Pickup, Feeding, and Chopping

Pickup and chopping require rapid regulation because windrow linear density and moisture are converted almost immediately into load variation. Windrow geometry provides feedforward information; feed-roll opening, pressure, cutterhead torque, and rotational speed provide feedback after entry. Their combination permits travel or feed-roll adjustment before large disturbances propagate.

Feedback corrects errors caused by uncertain bulk density, moisture, and compression. Overload and blockage protection take priority, followed by flow and particle-state regulation within downstream compaction, storage, and feeding constraints.

Feedforward and feedback signals must describe the same material parcel at compatible times. Geometry observed several meters ahead of the pickup predicts an approaching flow disturbance, whereas torque or pressure responds after material enters the machine. Delay compensation and coordinate alignment allow the controller to compare these signals without attributing the feedback response to the wrong windrow segment.

Chopping control distinguishes theoretical from actual particle size. Feed-roll speed, knife number, and cutterhead speed define the theoretical value, while moisture, orientation, wear, and shearbar clearance shape the distribution. Chen et al. [40] automatically compensated the clearance using vibration-based contact detection and position signals; Abilzhanuly et al. [79] showed that material alignment contributes to size dispersion.

Targets depend on use. In whole-plant maize, reducing processor-roll clearance improved kernel processing but reduced physically effective fiber, while theoretical length of cut mainly changed whole-plant and stover distributions [80]. Moisture, maturity, compaction, and feeding objectives therefore constrain the admissible particle state.

### 6.4. Intelligent Discharge Loading and Cooperative Vehicle Control

Continuous discharge couples material trajectory with trailer position, remaining capacity, pile distribution, and relative motion. Control seeks uniform capture and volume utilization without spillage or local overfilling.

Liu et al. [14] estimated trailer boundaries, pile contours, capacity, and the desired impact location. Trajectory control then compensates spout inertia, hydraulic delay, and vehicle motion [81]. Machine-level validation showed improved filling and reduced discharge loss [12].

Cooperative operation exchanges position, speed, heading, loading progress, and remaining capacity so that relative motion and vehicle replacement can be coordinated. State-machine and heterogeneous-planning studies provide transferable methods for task transition and resource allocation [82,83], although most grain-harvest evidence is methodological rather than direct forage-quality validation.

Immediate verification uses spout angle, predicted and observed impact position, trailer occupancy, and discharge loss. Remaining capacity is a moving boundary: a trajectory that is safe early in loading may create spillage or uneven compaction as the pile grows. Control therefore updates the target from both vehicle motion and the evolving material surface.

This control layer concerns the harvester and its associated transport vehicles; broader fleet scheduling and production-chain allocation are considered in Section 7.

### 6.5. Intelligent Baling, Wrapping, and Product-Formation Control

Baling control covers intake, formation, packaging, and discharge. Feed stability, density, geometry, moisture, and completion of tying or wrapping define the relevant state, while the density target depends on product pathway and storage risk.

Wang et al. [68] inferred density from roller reaction forces during formation, enabling immediate adjustment. Yin et al. [10] used dynamic weighing with attitude compensation to estimate mass and mean density at discharge, providing a finished-product record. The two measurements support fast regulation and later batch verification, respectively.

Moisture constrains density decisions. Dense hay at excessive moisture retains heat, whereas bale silage requires sufficiently uniform compaction for anaerobiosis. Changes in moisture or feed rate may require pressure, travel-speed, or target-diameter adjustment; high-risk moisture–density combinations remain attached to the bale record for storage monitoring.

Windrow position, left–right pickup loading, and chamber pressure can also correct transverse feed distribution before irregular ends and side-to-side density differences become fixed. Packaging completes the same sequence: automatic bale completion, net feeding and cutting, chamber opening, wrapping, and status recording preserve the state established during compaction [17,18].

Bale-level control also needs a stopping and classification decision. When target density or diameter is reached, the system must determine whether moisture, geometry, and formation history remain within the product boundary. A bale outside that boundary can be flagged for separate handling or intensified monitoring rather than treated as equivalent to the rest of the batch.

Across these operations, the value of sensing-driven control depends not only on actuator response but also on whether corrective action occurs before a state deviation propagates into a less reversible material condition. Immediate feedback is therefore most relevant to load, flow, geometry, particle formation, and product formation, whereas nutritive quality, fermentation, heating, and aerobic stability generally become available only after the effective intervention window has closed. These delayed measurements are better used for batch-linked verification, recalibration, and model updating than for direct real-time actuation. The remaining challenge is therefore to preserve state continuity across machines, operations, and time scales, which motivates the production-chain integration discussed in Section 7.

## 7. Production-Chain Integration, Key Challenges, and Research Priorities

The transition from intelligent machines to quality-oriented production is an integration problem. Material state evolves across operations, weather constrains the working window, capacities differ, and quality outcomes are delayed. Persistent batch identity and a spatiotemporal record link material, operations, environment, coordination events, and final evidence.

### 7.1. Production-Chain Integration and Comprehensive Evaluation

A minimum production record associates field and batch identity, time, product pathway, machine and implement, calibrated state estimates with uncertainty, key settings and events, and downstream sample or storage location. Georeferenced records can follow harvest-data standards [58]. Without continuity, final outcomes cannot verify upstream decisions.

Capacity differences also require coordination. Information exchange, task allocation, path coordination, and state synchronization [22,23] align mowing, conditioning, tedding, raking, baling, chopping, and transport. Independent throughput maximization can increase waiting or weather exposure even when each machine performs efficiently.

ISO 11783-1 and ISO 11783-10 support tractor–implement communication and task-controller exchange [84,85], but protocols alone do not ensure semantic continuity. Images, point clouds, spectra, load signals, quality estimates, and events need shared identities, definitions, time bases, spatial references, ownership, and governance [86].

Communication interoperability and semantic interoperability are distinct requirements. Hardware protocols can transfer data, but they do not by themselves define the meaning of material states, operation events, or relationships among material portions and batches. Cross-machine records therefore require shared definitions for moisture basis, windrow geometry, linear density, leaf-integrity proxies, particle state, bale density, and storage-risk variables; common event semantics for mowing, conditioning, tedding, raking, pickup, chopping, baling, wrapping, and drying, including settings, time, location, and operating condition; persistent material or batch identity; common temporal and spatial references; and metadata for calibration, uncertainty, units, measurement basis, and validity status. A forage-specific semantic layer is therefore needed above hardware communication protocols to support quality-oriented control and delayed verification. Within the literature examined in this review, a widely adopted model jointly representing material states, operation events, and persistent batch identity has yet to emerge.

Chain-level evaluation includes productivity, dry-matter recovery, quality, storage performance, energy, cost, and emissions. For intelligent systems, this evaluation must include both added system burden and realized operational benefit. Regional studies of dehydrated alfalfa and Chinese alfalfa production show that drying, irrigation, harvesting, and transport contribute differently to economic and environmental burden [87,88]. A quality gain is useful only when it justifies its resource and cost penalty.

A chain-level metric must preserve trade-offs rather than hide them in one score. Higher throughput may shorten weather exposure but increase fuel use or particle variation; intensified drying may preserve a harvest window while raising energy cost; denser products may reduce transport demand while increasing heat or effluent risk. Evaluation should report these component outcomes before applying any composite weighting.

### 7.2. Key Challenges

Six recurring gaps limit the transition from state observation to quality-oriented control. Table 6 links each gap to its control consequence, research priority, and required validation, providing a unified basis for the priorities discussed in Section 7.3.

The central weakness is causal continuity. Machine and proxy indicators often end the experiment before leaf retention, nutritive value, fermentation, or storage outcome is measured. External transfer is also uncommon: species, maturity, moisture, machine, season, and reference procedure shift the validity domain, so high accuracy in one dataset does not establish operational robustness [73].

Online forage-moisture sensing has been demonstrated using electrical, microwave, and spectroscopic approaches, and moisture information can in principle guide operating settings. Complete field-scale evidence is more fragmented because it must connect sensing, adaptive parameter adjustment, immediate material-state response, persistent assignment to the treated batch, and later verification of drying, fermentation, heating, or storage stability. Field evidence linking all of these interfaces within the same material batch remains limited. A similar discontinuity occurs for bale moisture and density sensing: compression or wrapping decisions may be adjusted, but the resulting density and sealing state are not always linked to later heating or aerobic stability. Most studies therefore validate individual sensing or machine-control links rather than the complete sensing–decision–actuation–quality pathway.

Broken batch records prevent delayed outcomes from updating earlier decisions, while cloud dependence introduces reliability and cybersecurity risks [70,89]. Short trials further underestimate wear, dust, drift, failure frequency, recovery time, and the economic or environmental cost of additional sensing and processing.

These gaps are coupled: external validation is difficult when event and batch identities are lost, causal analysis is weakened when final quality is not measured, and economic assessment is unreliable when benefits are inferred only from machine proxies.

### 7.3. Future Research Priorities

The priorities follow from the missing links, their consequences for control, and the evidence needed to resolve them.

Batch-linked experiments can connect mechanical action, material response, intermediate state, and final quality across contrasting materials and machines. Digital twins can then develop incrementally from validated local models, with new functions added after independent-batch tests show a defined decision benefit [90].

Transfer across seasons, sites, machines, and batches remains insufficiently established, particularly where uncertainty, out-of-domain behavior, and reference methods are incompletely reported. Reusable datasets can combine images, spectra, point clouds, machine signals, events, losses, and laboratory outcomes while preserving batch and temporal structure instead of relying on random partitions.

Reliable edge control depends on safety-critical functions remaining available during communication failure and on cross-machine records synchronizing when connectivity returns. Multi-season benchmarks are most informative when prediction performance is reported together with latency, drift, failures, recovery, fallback behavior, and manual takeover. System value can then be assessed jointly through quality, productivity, economics, energy, and environmental performance.

Cross-machine coordination is most defensible after local functions have defined fallback states and state-transfer semantics. A practical progression is to exchange validated batch and capacity records first, then coordinate rates and sequences, and finally update quality boundaries from delayed outcomes. This incremental route limits the risk that an unvalidated chain-level model propagates errors across otherwise reliable machines. A forage-specific semantic model that preserves shared state and event meanings with persistent batch identity across machines remains a priority.

Production-scale experiments are most informative when they preserve the chain between intervention and final outcome. A controller may reduce overload events yet increase exposure time, fuel use, or downstream waiting; a sensing model may remain accurate while fouling or maintenance requirements make continuous use impractical. Multi-season trials can report uptime, recalibration frequency, operator intervention, rejected or segregated product, and the proportion of batches with complete traceability alongside conventional prediction and machine-performance metrics.

Economic and environmental evaluation needs a counterfactual: the same production pathway under conventional operation. Added burden can be represented by hardware and integration costs, calibration and maintenance effort, and additional operator input. System availability, downtime, and fallback requirements describe reliability. Benefits include recoverable dry matter, avoided field or storage loss, labor and energy changes, rejected or segregated product, and quality-related value; changes in field passes and transport also matter where relevant. Reporting these components permits calculation of net benefit, payback period, cost per unit of dry matter handled or recovered, or avoided-loss value. These indicators show how farm scale, weather risk, and product premium affect feasibility without concealing shifts in cost or environmental burden between stages.

## 8. Conclusions

Mechanized forage production is path-dependent because each operation changes the material entering the next stage. Moisture distribution, leaf integrity, windrow geometry and linear density, mass flow, particle state, bale density, and pore structure explain downstream risk more directly than isolated machine-performance indices. Load, flow, geometry, particle formation, compaction, and safety can often be regulated from rapid measurements or proxy-state estimates. Nutritive quality, fermentation, heating, and aerobic stability develop more slowly and are better suited to batch-linked verification, recalibration, and model updating. Prediction accuracy contributes to control only when the estimate represents the affected material, remains valid under the operating conditions, and arrives while corrective action is still possible.

No universal harvesting optimum emerges from the literature. Outcomes vary with species, maturity, moisture, crop morphology, weather, machine configuration, throughput, sampling and reference methods, and the intended product. Hay, bale silage, and chopped silage therefore require different operating boundaries even when moisture, flow, geometry, or density values appear similar. Control must balance drying rate against leaf retention, throughput against particle or density uniformity, compaction against heating or effluent risk, processing intensity against energy demand and nutritive structure, and biomass recovery against contamination.

Implementation is limited by four related gaps. Complete batch-scale evidence seldom links sensing, decision, actuation, immediate material response, and final quality. Calibration and models often lack independent transfer across seasons, sites, machines, crops, and batches. Long-duration trials rarely characterize drift, fouling, wear, failures, uptime, and fallback operation, and cross-stage records do not consistently preserve batch identity. Techno-economic studies also provide limited evidence that added sensing and computation outperform conventional operation after installation, maintenance, energy, labor, and avoided-loss effects are considered.

Readiness falls into three levels. Level 1 includes machine load and feed rate, windrow position and geometry, discharge or loading position, material-flow stability, and bale-formation or mean-density proxies. These states are relatively mature for local short-loop regulation when sensors and actuators operate within validated domains. Level 2 includes moisture, spectroscopic composition, particle or chop state, and multimodal estimates. Their use remains condition-dependent because calibration drift, material presentation, crop and product differences, and transfer across seasons, sites, and machines can change performance. Level 3 includes leaf retention, final nutritive quality, fermentation quality, heating risk, aerobic stability, and other cross-stage outcomes. These delayed variables currently support batch-linked verification, supervisory control, model updating, and longer-horizon optimization rather than fast direct actuation.

Research should proceed in the same order as the evidence chain: establish representative state sensing; verify transfer with independent seasons, sites, machines, and batches; quantify state-to-action relationships; test actuator-level control in the field; connect interventions to batch-linked quality outcomes; maintain cross-stage data continuity and persistent batch identity; and evaluate production-scale economic performance. Improved prediction matters only when it changes a feasible decision and delivers a downstream benefit at an acceptable operational cost.

## Figures and Tables

**Table 1:** Operational risks, potential responses, and validation metrics for representative forage states.

Material State	Observable Indicators	Primary Risks	Adjustment Strategy	Outcome Verification
Wet, coarse-stemmed, or dense crop	Accumulation; feed-rate fluctuation; stem rebound	Blockage; under-conditioning; slow drying	Moderately reduce travel speed or roll clearance; improve aeration	Load variability; conditioning degree; stem–leaf moisture difference
Leaves dry before stems	Brittle leaves; high moisture variability	Leaf shatter during tedding; heating after baling	Reduce repeated disturbance; use a dew-recovery window; delay baling if needed	Leaf retention; moisture variability; bale temperature
Lodged or non-uniform crop	Abrupt canopy-height, orientation, or flow changes	Uncut forage; uneven swath; downstream feed shocks	Adjust height and speed by zone; stabilize swath width	Uncut loss; windrow linear density; peak load
Late maturity and greater lignification	Greater stem diameter and shear strength; lower leaf-to-stem ratio	Higher cutting energy and fragmentation; lower potential quality	Maintain knife sharpness; limit excessive speed and damage	Specific energy; fragment loss; leaf-to-stem ratio; NDF digestibility

Note: NDF, neutral detergent fiber.

**Table 2:** Diagnostic and traceability matrix for losses based on abnormal indicator combinations.

Abnormal Indicator Combination	Most Likely Stage	Discriminating Evidence	Potential Confounders	Control Implication
Lower dry-matter recovery with little change in leaf-to-stem ratio	Mowing, pickup, or discharge	Spatial pattern of field residue; uncut and pickup losses	Within-field yield variability	Identify incomplete coverage or material-transfer loss
Lower leaf-to-stem ratio and CP; higher NDF and ADF	Tedding, raking, or pickup	Leaf retention and proportion of fine fragments	Maturity and initial leaf-to-stem ratio	Identify selective leaf loss and reduce disturbance
Higher ash without marked dry-matter loss	Raking or pickup	Ground clearance; soil particles; spatial ash pattern	Initial ash concentration	Adjust rake/pickup height and travel path
Heating or abnormal pH with lower aerobic stability	Baling, wrapping, or storage	Incoming moisture; density uniformity; seal integrity	Initial sugar concentration and inoculant	Distinguish moisture, compaction, and sealing failures

Note: CP, crude protein; NDF, neutral detergent fiber; ADF, acid detergent fiber.

**Table 3:** Primary decisions, dominant trade-offs, and quality boundaries for major forage-product pathways.

Product Pathway	Primary Decision Point	Dominant Trade-Off	Priority Coordinated Strategy	Non-Negotiable Boundary
Hay	Tedding, raking, and baling window	Drying rate versus leaf retention	Coordinate tedding, raking, and baling using moisture and forecast	Do not bale above the safe moisture range; limit disturbance after leaf brittleness develops
Bale silage	Raking–wrapping window	Field loss versus fermentation moisture	Coordinate compression, bale diameter, and wrapping with moisture and feed rate	Avoid inadequate density, film damage, and delayed wrapping
Chopped silage	Feeding, chopping, loading, and packing	Capacity versus particle state and compaction	Match travel, feeding, chopping, discharge, and packing rates to material flow	Avoid sustained overload, uncontrolled particle-state variation, and excessive discharge loss

**Table 4:** Cross-stage propagation of operational abnormalities, latest compensation windows, and end-point verification.

Upstream Abnormality	Downstream Propagation	State Available for Advance Detection	Latest Compensation Window	End-Point Verification
Swath too narrow or thick	Slow internal drying → additional tedding → leaf-loss or rain risk	Swath width, thickness, and moisture gradient	Before the first tedding pass	Drying uniformity and leaf retention
Under- or over-conditioning	Slow drying or leaf/effluent loss → collection and storage risk	Conditioning degree, mat thickness, and load	Before the next operation	Stem-leaf moisture difference and dry-matter recovery
Variable windrow linear density	Unstable pickup feed → variable particle size or density	Mass per unit length and machine load	Before pickup or chopping	Particle-size CV and bale/silo density CV
High and non-uniform moisture	Incomplete air exclusion → heating, mold, or undesirable fermentation	Mean and variance of moisture; temperature and humidity	Before baling or ensiling	Core temperature, pH, and aerobic stability
Seal or film defect	Oxygen ingress → secondary fermentation and dry-matter loss	Film integrity, pressure decay, and surface temperature	Before leaving the wrapper or completing sealing	Leak location, heating, and mold incidence

Note: CV, coefficient of variation.

**Table 5:** Representative sensing combinations, reported evidence, and deployment requirements for control-relevant forage states.

Control-Relevant State	Representative Sensing Combination	Reported Performance Evidence	Major Confounding Factors	Deployment Requirement
Moisture and drying state	Electrical or NIR moisture sensing + weather and windrow information	Portable NIR achieved R^2^ = 0.985 in a small moisture dataset [49]	Density, temperature, contact condition, stem–leaf differences, product form	Independent-material calibration; response-time evaluation; validation across extreme moisture conditions
Feed load and mass flow	Feed-roll displacement, impact force, pressure, or torque + moisture and travel speed	Sensor fusion reduced SEE to 0.3 kg s^−1^ [56]; independent validation achieved RMSE = 2.62 kg s^−1^ [47]	Dynamic impact, material compressibility, moisture variation, machine settings	Transport-delay correction; dry-matter mass balance; validation across operating conditions
Particle/processing state	High-speed RGB or hyperspectral imaging + feed rate, moisture, and machine settings	KPS: r(38) = 0.98 versus sieving [63]; hyperspectral kernel classification [62]	Motion blur, overlap, crop type, moisture, illumination	Standard reference; representative sampling; latency; cross-crop validation
Windrow geometry and operating scene	RGB/depth imaging or LiDAR + machine pose and travel speed	LiDAR point clouds provided windrow position, orientation, and field mapping [59]	Illumination, dust, occlusion, discontinuous windrows, terrain variation	Processing-latency assessment; spatial alignment; recovery from perception failure
Nutritive quality and material composition	NIR or hyperspectral sensing + moisture, cultivar, and harvest-cycle information	Fresh-forage dry matter reached R^2^p = 0.96 [64]; customized handheld models achieved r^2^ ≥ 0.90 for selected quality traits [67]	Water absorption, scattering, sample presentation, instrument variation	Independent-batch validation; calibration transfer; drift detection; uncertainty and reference-laboratory agreement
Bale formation state	Dynamic weighing, compression force, dimensional sensing + moisture and film-state monitoring	Process density prediction accuracy reached 93–97% [68]	Internal moisture/density gradients, bale geometry, incoming feed variability	Spatial-uniformity assessment; abnormal-bale detection; validation against subsequent heating or storage failure

Note: Reported R^2^, r^2^, RMSE, SEE, SEP, and accuracy values were obtained with different reference methods, data partitions, material ranges, and units and are not directly comparable. KPS, kernel processing score; R^2^/r^2^, coefficient of determination; RMSE, root mean square error; SEE, standard error of estimate; SEP, standard error of prediction.

**Table 6:** Control-limiting research gaps and the validation required for quality-oriented forage production.

Current Gap	Why It Limits Control	Research Priority	Required Validation
Weak machine–quality causal linkage	Improved proxies may not preserve final quality	Batch-linked causal experiments	Batch-linked field experiments with final nutritive, fermentation, heating, and storage outcomes
Poor sensing transferability	Models may fail across crop, machine, or season	External validation and out-of-domain detection	Independent seasons, sites, machines, and batches
Broken cross-stage data continuity	Downstream outcomes cannot update upstream decisions	Persistent batch identity and event records	Full-chain traceability
Reliability and connectivity risks	Critical control cannot depend on continuous cloud access	Edge control, conservative fallback, and recovery logic	Fault-injection and recovery tests
Limited production-scale evidence	Short trials underestimate drift, wear, and failure	Long-term field benchmarks	Multi-season deployment
Uncertain economic and environmental benefit	Added intelligence may not yield a net benefit	Multi-objective techno-economic evaluation	Hardware/integration cost; calibration/maintenance burden; uptime/downtime; energy, loss reduction, quality value, and net benefit

## Data Availability

No new data were created or analyzed in this study. Data sharing is not applicable to this article.

## Abbreviations

The following abbreviations are used in this manuscript:

ADF	acid detergent fiber
ASABE	American Society of Agricultural and Biological Engineers
CNKI	China National Knowledge Infrastructure
CP	crude protein
CV	coefficient of variation
GNSS	Global Navigation Satellite System
ISO	International Organization for Standardization
KPS	kernel processing score
LiDAR	light detection and ranging
NDF	neutral detergent fiber
NIR	near-infrared
NIRS	near-infrared spectroscopy
RGB	red–green–blue
RMSE	root mean square error
RMSEP	root mean square error of prediction
SEE	standard error of estimate
SEP	standard error of prediction
SWIR	short-wave infrared
